# **Doctor Retention or Migration: From Ireland to the World?** Comment on "Doctor Retention: A Cross-sectional Study of How Ireland Has Been Losing the Battle"

**DOI:** 10.34172/ijhpm.2020.196

**Published:** 2020-10-20

**Authors:** John Connell

**Affiliations:** School of Geosciences, University of Sydney, Sydney, NSW, Australia.

**Keywords:** Doctors, Migration, Workplaces, Livelihoods, Ireland, Policy

## Abstract

The recent study of prospective doctor migration and retention suggests that more than half of junior doctors intend to migrate from Ireland. While intent is not necessarily outcome, such intentions match similar survey results in Ireland and elsewhere. The rationale for migration is described as a function of difficult workplace circumstances (notably long hours and mismanagement). Lifestyle factors may however also be important for both migration and significant levels of return migration. These are related to family formation, and to an established culture of migration, that has contributed to a considerable circularity of mobility and migration, primarily between Anglophone countries. International migration may also have unspecified regional variations and impacts. Migration has taken a similar form for half a century and longstanding policies to constrain its more damaging impacts have been conspicuously unsuccessful yet responses remain urgent.

Brugha et al^1^ provide a valuable quantitative analysis of the probability of migration of junior (mainly therefore young) doctors from Ireland following graduation. It is a valuable addition to the considerable number of studies of the migration of skilled health workers, enabling a well-documented consensus on the significance of workplace problems and other issues in migration from Ireland.^2^ The study reveals that more than half of junior doctors in Ireland have stated that they are likely to leave the Irish workforce within, presumably, the next few years (since no date was specified), although two thirds of these expressed a wish to return to work in Ireland at some point. Those who are most likely to migrate are relatively young (<30), non-European Unions (EU) nationals, and concerned at weak supervision, high training costs and inadequate supervision. Women are less likely to wish to migrate.

Intentions are not realities, and frustrations over many problems are often expressed in terms of mobility, but nonetheless such intentions generally correlate with and emphasize the conclusions of previous studies in Ireland that have pointed to similar migration outcomes, and where migration has come to fruition.^3^ In a country once again characterized by emigration this should continue to be so. However perhaps the most positive aspect of the survey is that only 3% of all young doctors intend to give up entirely and drop out.

 While 52% of all graduates may eventually be lost to Ireland – at least temporarily- they are not lost to the profession (or at least there is no evidence of that). While most migrate to other Anglophone countries and over time are more likely to stay there, those who are non-EU nationals conceivably intend to return to countries with lower doctor: population ratios and more significant healthcare needs. It would be useful to know.

## Globalization

What is true of Ireland is true of many other countries in most world regions. In some respects the most significant thing that distinguishes this study from many others is the sophisticated and substantial quantitative analysis. Sadly, for policy formation, other than the need to focus on the requirements of particular specialisms, there is nothing here that is particularly new. Brugha et al themselves point to similar contemporary European structures of migration – rationales and proportions – for Portugal,^4^ Germany,^5^ Romania, Greece and the United Kingdom. To those can be added Lithuania^6^ and Iceland.^7^ The proportions that wish to leave are generally highest in relatively poor countries, and lowest in the richest; however Iceland proves an exception, with 63% at least ‘considering’ migration whereas in eastern Germany the similar figure was 30%. As in Ireland, interest in migration increased following recession, as livelihoods seemed threatened. A very similar pattern of migration of doctors is true of such Anglophone nations as Canada, Australia and New Zealand.^8^

Rationales for migration are almost identical, accentuated in slightly different ways: unsatisfactory working conditions (whether expressed as poor work-life balance, long hours, inadequate access to technology, poor supervision, inadequate salaries …) and perceptions of superior conditions (and experience and salaries) elsewhere. Most of the differences between countries are quite subtle and emphases are as much a result of how surveys were undertaken as much as real differences. Nor are these circumstances new; they have been documented over a much longer time period, and are also broadly those that have stimulated the migration of nurses, pharmacists and other health professionals. Moreover, in Ireland as elsewhere, the reasons given for intended migration generally correlated with the reasons for migration given by doctors who had already migrated: working conditions, training opportunities and poor career prospects.^9^

 Significantly almost 90% of those who intended to move intended to move to another Anglophone country – led (but only just) by near neighbour the United Kingdom. Ironically Irish junior doctors are migrating to Australia, as Australian (and New Zealand) junior doctors are moving out, and for the same reasons. Similar patterns thus exist in most Anglophone contexts as doctors (and other skilled workers) move between them, as the outcome of similar structural contexts and with similar professional and also, significantly, personal aspirations.

 Most similar studies in Anglophone countries (other than the United States) point to a considerable circulation, suggesting that mobility is related to lifestyle migration. Indeed the dominance of the United Kingdom (sometimes subsumed as ‘London’), and even of the four largest cities as the destinations of Irish doctors in Australia, point to the association between lifestyles and professional aspirations.

What is distinctive about Ireland is that in modern times it has always been an exporter of doctors, part of a much wider labour-exporting context. Thus Oscar Gish documented an already established migration of Irish doctors in the 1960s and, a decade later, the World Health organization (WHO) singled out Ireland as ‘an exceptional case in that it is an exporter’ of doctors.^10^ Remarkably, in almost every similar subsequent survey, and perhaps disturbingly, the same factors have tended to recur. The only distinction is that the Anglophone world, other than the United States, has now ‘caught up’ with Ireland, as other countries produce their own flows, and face similar problems.

Over the same half century there has been a growing and very well-documented export of skilled health workers from the Global South that indicates many similar issues to those identified by Brugha et al (lack of access to technology, poor supervision, mismanagement and overwork …) but usually more extreme and perhaps with the critical distinction of more inadequate salaries. Well-documented flows have been analysed from certain impoverished nations, such as Zimbabwe, and especially from sub-Saharan Africa and small island states.^11^ That contributed to problematic replacements in Ireland from the Global South. Migration has left giant gaps in healthcare systems especially in rural and regional areas, and resulted in considerable disappointment and scepticism over the ability of the WHO Global Code to discourage international migration. The mantra ‘train and retain’ is insufficient, not only in Ireland, but is less problematic there than in sub-Saharan Africa, despite being potentially unsustainable and posing severe costs.^12^

## Space, Place, Livelihoods and Culture

 Brugha et al do not report on possible regional variations so we do not know if doctors were more likely to seek to migrate from Dublin or from smaller cities and regional centres. (It is not evident whether such a location question was asked; if it was asked, the resultant analysis would be useful). Ubiquitously countries find it more difficult to provide and support human resources in rural and regional areas.

 In most contexts such spatial variations are significant, firstly, because many perceive that general amenities and life chances are superior in larger cities. These may be social, cultural or technical (and even as simple as where relatives live). Secondly, because most doctors are married (albeit no more than at best 60% in this study), and often to other skilled workers, so that the task of finding jobs for partners in smaller places is particularly difficult. Partners themselves may experience the same frustrations in their own sectors (often education) that doubles the demand for mobility; they may even be the more influential decision-makers. Thirdly, doctors aspire to a good education for their children and that is normally more readily available in larger places. (That also implies that policies to attract and retain health workers must consider households). Frustratingly the possibility of influences on migration coming from outside the professional context of medicine were absent (although basic data were collected on whether respondents had partners and dependents).

 The ages at which doctors stressed that they were most likely to migrate (here in their late twenties and early thirties) ties in with decisions quite probably being taken as much for (young) family reasons as for purely professional reasons. It is impossible to tell. The implication is that potentially mobile doctors are most likely to be younger doctors in the process of early family formation. Indeed, and not only for doctors, this is the time in life when people are most free to move before more comprehensive and demanding family and other responsibilities eventuate.

In studies in destinations it is evident that doctors who have migrated tend to stay and postpone or abandon return migration, and that the window of time for return declines over time^13^ for family reasons rather than professional ones. Indeed Brugha and colleagues’ survey data show that those with dependents were much more likely to intend to remain in Ireland.

 This does not necessarily imply that doctors (and others) must migrate internationally rather than nationally, but it does imply that health facilities and management structures may not necessarily be the primary instigating factor in migration, however crucial they seem. Nor does that deny the need to improve the context to make return migration attractive, although doing so becomes more complex.

## Policy Implications

Given the significance of emigration in Ireland (relative for example to most other west European nations) it is unsurprising that it became of particular concern during and following the 2008 collapse of the Celtic Tiger economic boom, but less well-documented in other highly skilled sectors. Doctors are certainly not the only skilled (or otherwise) migrants leaving Ireland. There too migration is related to lifestyle considerations as much as to professional circumstances.^14^ Brugha and colleagues’ paper, like so many others, is part of that legacy. It is important to ask how structural circumstances might reasonably be changed to stem such wide-ranging flows, even within a discourse around an ideology and reality of return.

In this context migration is normative - expected and anticipated in equal measure – across the board – even a rite of passage within a ‘culture of migration’ and not easily, or at all, to be discouraged and confronted.^15^ Overseas experience is valued for more than simply technical reasons as a boost to career progression. That enables a better understanding of the diverse migration dynamics. While many professionals migrate at some point in their lives, that is not necessarily problematic; many return, and those who do may bring back superior skills, valued experience (and even capital). Employment practices suggest this is so.

 Brugha et al reach what amount to familiar policy conclusions that centre on inadequate working experiences. They indicate the problems of poor training, excessive demands, and stress (and even bullying) and thus point to the necessity of diverse retention strategies. Ironically, the first conclusion is the need to remedy understaffing and beyond that to facilitate circular migration, and to make allowances and contingencies for particular specialisms. With some exceptions such policy implications have been unchanged for decades, and not only for Ireland. As Brugha et al well recognise, it is of considerable concern that despite several reports on medical workplaces and training needs, and the drawing up of national retention measures, they have ultimately failed to resolve basic stressful working conditions and unsatisfactory training, or staunched substantial migration. But, as lifestyle factors indicate, that is never wholly possible. Nor are structural circumstances easily overcome. It is equally dispiriting that these problems emanate from the inability of adequate financial resources to be dedicated to the health sector – a rather similar but less extreme version of that in the Global South – but which then amplifies migration.

## Conclusion

Mobility is engrained in contemporary life. That will not change. Brugha and colleagues’ study emphasizes long established issues of training and management. Ultimately these are only resolved by more adequate public funding. In a contemporary professional environment it is worrying that bullying remains a problem in workplaces, and that remarkably little has changed in half a century. Brugha et al quote Gish, writing in 1969, on the rationale – ‘lack of advancement, the desire for further specialization, for better pay’ while the policies he suggested remain appropriate. But, beyond that, Gish suggested a superior education, manpower [sic] and employment strategy that would combine income incentives, promotion prospects and ‘higher levels of support from the state’ alongside proper infrastructure, more rapid promotion, and more flexible salary scales.^16^The more things change … the more that austerity is no assistance to retention. Indeed the health system faces very similar issues to those of the higher education system and, rather closer and now more pertinently, the nursing home sector.^17^ Yet, in the age of coronavirus disease 2019 (COVID-19) it is surely possible, above all, to provide better workplace experiences for all health workers, and ensure a superior ‘new normal.’ As everywhere else, Irish doctors will continue to migrate (usually with their families) but they will need to be assured of a warm welcome when they return,^18^ amidst the ever optimistic belief that all health workers should be valued much more highly.^19^ The room to manoeuvre within neo-liberal political economic systems greatly limits the scope for effective changes yet equity and empathy demand nothing less. It does not however seem particularly likely as COVID-19 era politicians focus on renewing old economic systems.

## Ethical issues

 Not applicable.

## Competing interests

 Author declares that he has no competing interests.

## Author’s contribution

 JC is the single author of the paper.
